# Venezuelan equine encephalitis virus: novel live-attenuated vaccines for inducing complete protective immunity

**DOI:** 10.1038/s44298-026-00186-5

**Published:** 2026-03-21

**Authors:** Kenneth C. Elliott, David Saunders, Joseph J. Mattapallil

**Affiliations:** 1 Dept. of Microbiology and Immunology, Henry M Jackson Foundation for Military Medicine, Uniformed Services University, Bethesda, MD USA; 2 Translational Medical Unit, Dept. of Medicine, Uniformed Services University, Bethesda, MD USA; 3 Dept. of Microbiology and Immunology, Uniformed Services University, Bethesda, MD USA

**Keywords:** Diseases, Immunology, Microbiology

## Abstract

Venezuelan equine encephalitis virus (VEEV) is a mosquito-borne alphavirus that causes severe neuroinflammation and fatal infections in some people. Numerous outbreaks of VEEV have been reported in Latin America in the past century. Though mosquito-borne, studies have demonstrated that aerosolized VEEV infections lead to significantly higher mortality rates in animal models, suggesting that VEEV, if aerosolized, could cause widespread infections. There are currently no FDA-approved vaccines against VEEV, though TC-83, a live-attenuated strain of VEEV, has been tested as an investigational new drug in laboratory personnel and at-risk health workers. Its use, however, has been associated with severe adverse events and variable immunogenicity. Novel live-attenuated vaccines such as the V4020, V3526, VRC-WEVVLP03-00VP, 68U201/IRES1, and others are under development to overcome some of the limitations associated with the TC-83 vaccine. Here, we discuss the pathogenesis of VEEV and the current state of VEEV vaccines that are in clinical development.

## Introduction

Venezuelan equine encephalitis virus (VEEV) is a re-emerging alphavirus that causes severe disease in humans and animals^1^. Large numbers of infections in both humans and equines (horses, donkeys, and mules) have been extensively documented, with a significant potential for future outbreaks^2–4^. Despite this, there are currently no effective vaccines or treatments to prevent or control outbreaks. VEEV transmission includes an endemic and an epidemic cycle, with *Culex* mosquitoes and small mammals involved in endemic transmission, whereas the transmission and circulation of epidemic VEEV to humans and horses are primarily driven by *Aedes* mosquitoes^5–9^.

The first major outbreak of VEEV was reported in horses in Colombia and Venezuela between 1936 and 1943. While anecdotal reports of human infections date back to 1952, official documentation of human fatalities was not available until the 1962–1972 outbreak^2^. The outbreaks between 1962 and 1972 included a series of massive epizootic and epidemic outbreaks that started in La Guajira, Colombia, in 1962 and then spread across countries in South and Central America, Mexico, and the continental US. Since then, the largest outbreak of VEEV in humans was reported in 1994–1995, during which an estimated 100,000 people were infected in Colombia and Venezuela, along with many horses. Although the fatality rates in humans were less than 1% during the 1995 outbreak, an estimated 8% of Colombia’s entire horse population is believed to have died from infection, raising the possibility of a wider spillover to humans^10^. Other studies in preclinical models suggest that aerosolized VEEV could lead to significantly higher mortality rates than mosquito-transmitted infections^11,12^. While VEEV outbreaks in the past have been primarily due to spill over into human and equine populations, the virus’s stability, low infectious dose, and potential for aerosolization have raised significant concerns about its ability to cause widespread infections worldwide. Other equine encephalitis viruses, such as Western equine encephalitis virus (WEEV) and Eastern equine encephalitis virus (EEEV), have also been shown to cause CNS pathology in experimental animal models following aerosol challenge^13–16^. Given its potential to rapidly spread, the National Institute of Allergy and Infectious Diseases (NIAID) designated VEEV as a biodefense priority pathogen, underscoring the need for the development of efficacious vaccines and countermeasures. Early attempts led to the development of the TC-83 and C-84 vaccines. The TC-83 is a live-attenuated VEEV vaccine used as an investigational new drug that was recommended for use in laboratory workers and at-risk personnel working with the wild-type virus^17^. Since its initial use, the TC-83 vaccine has been associated with severe adverse reactions and a lack of seroconversion in up to 20% of vaccinees, which, along with the potential for reversion, has raised significant concerns over its continued use as a vaccine^17–20^. Unlike the live-attenuated TC-83 vaccine, the C-84 vaccine is a formalin-fixed version of the TC-83 vaccine, which was, however, found to be poorly immunogenic in humans^17^. There are currently no FDA-approved VEEV vaccines for use in humans or animals, though several are in preclinical or early clinical trials. In this review, we provide an in-depth look at VEEV and the novel vaccine strategies that are currently under clinical development.

## Epidemiology

**Fig. 1:**
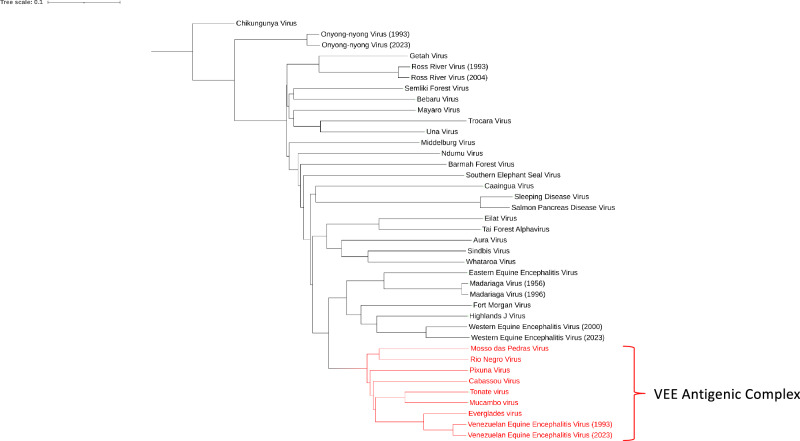
*Alphavirus* phylogenetic tree based on full genome sequences. The phylogenetic tree was created by obtaining complete nucleotide reference sequences from NCBI Virus. Clustal Omega “Multiple Sequence Alignment” was used to align sequences, and the Clustal Omega “Simple Phylogeny” was used to calculate distances and generate the phylogenetic tree. iTOL Interactive Tree of Life was used to visualize the tree and add color to highlight the VEE Antigenic Complex (Red).

VEEV is a member of the *Togaviridae* family and belongs to the *Alphavirus* genus that includes chikungunya virus, WEEV, and EEEV (Fig. 1). The VEEV antigenic complex (VEEAC; Fig. 1) is comprised of a group of closely related alphaviruses that are grouped into 6 antigenic subtypes, namely, I–VI, that cause encephalitis-like disease in humans and horses^1^. VEEV belongs to the antigenic subtype I that includes 5 variants, namely, IAB, IC, ID, IE, and IF, with the other alphaviruses belonging to the antigenic subtypes II (Everglades virus), IIIA (Mucambo virus), IIIB (Tonate virus), IV (Pixuna virus), V (Cabassou virus), and VI (Rio Negro virus). The VEEV subtypes IAB and IC were primarily responsible for the major outbreaks in humans and horses, with the other subtypes largely restricted to rodents and mosquitoes^1,2,20^. Horses typically act as amplifying hosts for VEEV IAB and IC subtypes, whereas humans are considered dead-end hosts for any VEEV type. VEEV IAB and IC produce high viremias in horses that promote further mosquito transmission of the virus in epidemic/urban cycles. Interestingly, unlike VEEV, horses are not seen as amplifying hosts for WEEV and EEEV^21^. Studies have shown that subtypes IAB and IC likely descended from the ID subtype, whereas subtypes IE and IF were distinct from this group^22^. Anishchenko et al. reported that the IC subtype of VEEV that was responsible for the 1992–1993 outbreak in western Venezuela emerged from an ID subtype through a series of mutations, whereas a single mutation in the E2 protein from Thr → Arg at position 213 was sufficient for enhancing virulence and replicative capacity in horses^23^.

**Fig. 2:**
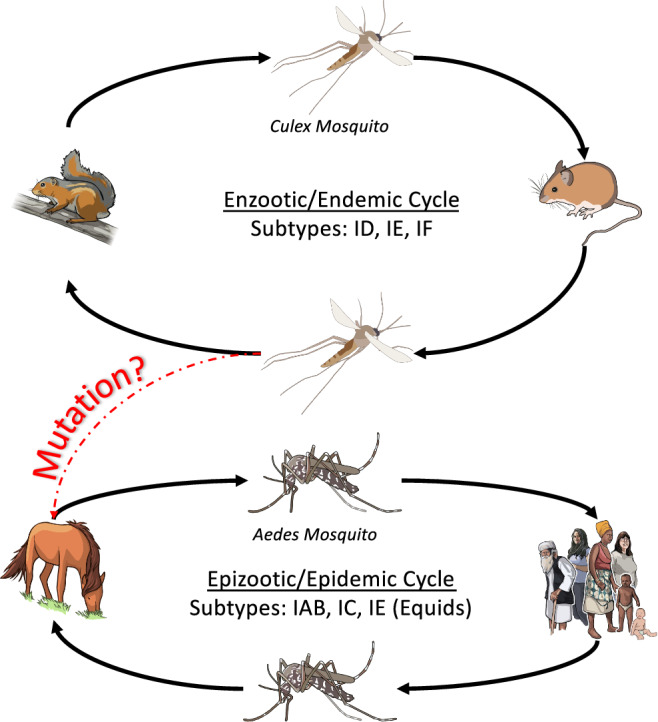
Basic outline of the VEEV host cycle. The enzootic/endemic cycle is continuous in the wild, typically circulating between small mammals and *Culex* mosquitoes. The spill over into an epizootic/epidemic cycle is hypothesized to occur when mutations occur in the endemic strains that allow for infection of *Aedes* mosquitoes, humans, and equids. Images used were obtained from the NIH BIOART source.

VEEV transmission includes an endemic cycle in which the virus circulates between *Culex* mosquitoes (*Cx. portesi, Cx. cedecei, Cx. vomerifer, Cx. pedroi, Cx. taeniopus*, *and Cx. adamesi*) and small rodents such as the spiny rats (Proechimys spp.), cotton rats (Sigmodon spp.), and rice rats (Oryzomys spp.)^5,7,9^, and an epidemic cycle in which VEEV circulates between infected *Aedes* mosquitoes, humans, and horses (Fig. 2). Interestingly, *Aedes* mosquitoes (*Ae. taeniorynchus and Ae. sollicitans*) serve as the primary vectors for VEEV transmission to humans and horses during epidemic cycles, suggesting that specific VEEV subtypes have likely evolved strategies to maintain their lifecycle within *Aedes* mosquitoes^8^. In particular, the PE2 gene has been shown to have a modest effect on the ability of VEEV subtypes to infect *Aedes* mosquitoes^6^. Unlike the *Culex* and *Aedes* mosquitoes, the *Psorophora* genus of mosquitoes (i.e., *Psorophora columbiae*) was shown to be susceptible to both endemic (IE) and epidemic (IAB) subtypes, suggesting that *Psorophora* mosquitoes may serve as an important link between the endemic and epidemic cycles^24,25^. Though both endemic and epidemic subtypes of VEEV can cause severe disease in horses and humans, only the epidemic strains have been shown to replicate efficiently enough in horses to sustain the epidemic cycle.

Outbreaks of VEEV have been frequently documented in both humans and horses, dating back to 1936 and as recently as 1996^3,4^. The first recorded outbreak occurred in Colombia between 1935 and 1943. Another outbreak was reported between 1942 and 1946 in Peru and Ecuador, largely restricted to horses. The first human cases of VEEV infection were officially diagnosed in Colombia in 1952 due to the prevalence of human encephalitis cases. Less than a decade later, a short-lived outbreak was reported in Colombia and Venezuela between 1962 and 1964 that was the first reported large-scale infection with human fatalities numbering over 25,000 confirmed cases and 156 deaths. Following this outbreak, an epidemic VEEV outbreak occurred between 1969 and 1972 that started in the border areas of Guatemala and El Salvador and spread north into Mexico, with over 50,000 fatalities in horses and 93 fatal cases in humans^2^. During its peak, the outbreak spread to horses and humans in Texas, though fatalities were primarily restricted to horses^26^. A smaller outbreak in western Venezuela occurred between 1992 and 1993 that preceded the large-scale outbreak of 1995, which resulted in ~75,000 reported human cases with 300 deaths, along with an estimated loss of 8% of Colombia’s equine population that was thought to be driven by an increase in vector densities associated with rainfall and humid conditions^10^.

## Structure and replication

**Fig. 3:**
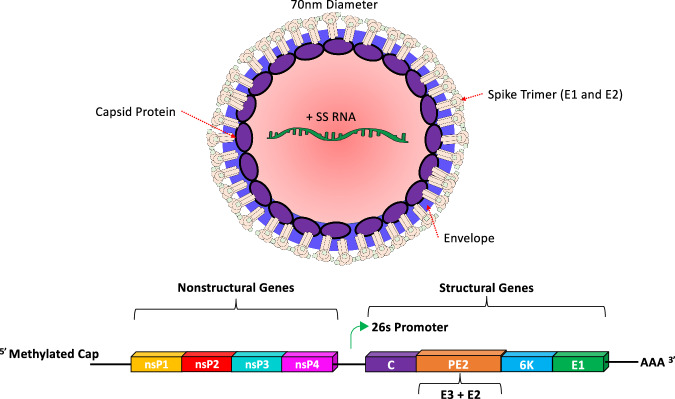
Cartoon drawing of VEEV virion and genome structure. VEEV is part of the *Togaviridae* family and the *Alphavirus* genus. The positive-sense, single-stranded genome is ~11.5 kb long and encodes 4 non-structural and 5 structural proteins. Only three of the structural proteins (C, E2, E1) are thought to be represented in an individual virion, although there is some evidence of E3 being present in small amounts. Typically, E3 serves as a chaperone for E2 and is cleaved off in the Golgi during processing. The 6K protein is a viroporin that plays a key role in virus assembly and budding. The nonstructural proteins have a wide variety of functions during replication: nsP1 caps the viral genome; nsP2 cleaves the viral polyproteins; nsP3 acts as a scaffolding protein and contributes to the formation of the viral replication complex; and nsP4 is the RNA-dependent RNA polymerase.

VEEV is an enveloped, positive-sense, single-stranded RNA virus with an ~11.5 kb genome that encodes 4 nonstructural genes, nsP1–nsP4, and 5 structural genes, E1, E2, E3, 6K, and C (Fig. 3). Infection is initiated when the surface glycoprotein E2 binds to the LDLRAD3 (Low-Density Lipoprotein Receptor Class A Domain-Containing 3) receptor^27–29^ on the surface of host cells.

In the cell cytoplasm, viral genome expression starts with the translation of the polyprotein p1234, which is cleaved by the viral protease nsP2 into nsP4 and p123^30–32^. The nsP4 and p123 together form the early replication complex to synthesize the negative-sense RNA strands^33^, whereas positive-sense RNA synthesis occurs when all 4 nsps are cleaved. Negative-sense strand synthesis partially stops once nsP1 is cleaved, and the remaining nonstructural proteins, p23, and nsP4, transition to form the initial replication complex, where negative-sense RNA continues to be synthesized alongside positive-sense RNA. Cleavage of p23 to nsP2 and nsP3 leads to the maturation of the replication complex, where only positive-sense genomic and subgenomic RNAs are synthesized.

Within the replication complex, the nsP1 protein is responsible for capping the viral genome and protecting it from cellular nucleases^34^, nsP2 acts a viral cysteine protease that cleaves the translated nonstructural polyprotein into individual units^35^ and plays a role in assembling the VEEV replication complex^36^, nsP3 acts as a scaffolding protein that associates with other nonstructural proteins to form the viral replication complex^32,37^, and associates with cellular proteins to form cytoplasmic complexes that aid in infection^38^. The nsP4 protein is the viral RNA-dependent RNA polymerase that mediates the synthesis of the positive and negative strands of the viral genome, which is essential for VEEV to replicate effectively^39^.

The subgenomic RNA that encodes the structural proteins C, E3, E2, 6K, and E1 is translated into a polyprotein. Following autocatalytic cleavage of the newly synthesized capsid, the envelope proteins E1 and E2 undergo post-translational modification in the endoplasmic reticulum (ER) and are expressed on the cell membrane as trimeric spikes^40–42^. Interestingly, the E2 protein is synthesized from the precursor protein PE2, which contains both the E2 and E3 proteins (Fig. 3), and is proteolytically cleaved in the trans-Golgi by furin to generate mature E2 and E3 proteins^41,43^. Though the exact function of E3 is not clear, the 7 kDa E3 glycoprotein is thought to stabilize the E1–E2 heterodimer in the Golgi prior to the release of virions by budding^44^. Others have suggested that E3 likely serves as the signal sequence for E2^45^ or may be secreted from infected cells^43,46,47^. Although the role of E3 in viral replication still needs to be clarified, induction of E3-specific antibodies, though non-neutralizing, has been shown to suppress VEEV replication and protect mice from lethal challenge, suggesting a role for E3 in viral pathogenesis^48^. The capsid assembles as a nucleoprotein complex in the cytoplasm that encases the genome before release of intact, mature virions from the cell surface by budding^40–42^. The capsid also plays an important role in pathogenesis by inhibiting the transport of various host proteins into the nucleus and blocking cellular transcription, which, in turn, leads to cell cycle arrest^49^. During the budding process, the 6K protein functions as a viroporin, which plays a key role in virus assembly and budding from the infected cells^50^.

## Pathogenesis

Infection is usually characterized by acute viremia that peaks between days 2 and 3 and lasts for a week. VEEV has been shown to infect myeloid cells, such as dendritic cells and macrophages, allowing it to spread to and replicate within local lymphoid tissues^51^. Replication in the lymphoid tissues is accompanied by rapid viremia followed by systemic dissemination to the CNS either through retrograde neuronal transport^52^ or through the blood-brain barrier^53,54^. Charles et al., using mouse models, reported that VEEV could infect the CNS via the olfactory bulb and the trigeminal nerve^55^, suggesting that multiple mechanisms may play a role in VEEV entry into the CNS.

Initial infection is associated with febrile illness, including fever, myalgia, chills, mouth ulcers, vomiting, sore throat, leukopenia, and diarrhea, within 1–4 days after infection, which usually resolves within a week. However, more serious neurological complications have been reported primarily in children and older adults^56^ with CNS-related symptoms lasting up to 6 weeks after onset of infection^2^. Due to limited surveillance, the number of asymptomatic and undiagnosed cases is hard to determine, though some studies suggest that VEEV infections likely account for up to 10% of cases diagnosed as dengue in Latin America^57^.

The neurological symptoms described during the 1995 Colombian outbreak included confusion, hallucinations, gait abnormalities, hemiparesis, acute convulsions, focal seizures, stupor, encephalitis, coma, and, in some cases, death. In fatal human cases of VEEV, pathologies were reported in the lungs, lymphoid tissues, and liver with CNS lesions that included edema, congestion, hemorrhage, vasculitis, and meningitis/encephalitis^58,59^. During the peak of the 1995 Colombian outbreak, a significant increase in spontaneous abortions was reported during the month of September at the Riohacha hospital in La Guajira city, where the average number of abortions/month was twice the monthly average that was recorded between 1992 and 1995 prior to the outbreak; 40 abortions/month as compared to ~18 abortions/month^10^. Wide-spread testing data are not available, likely due to limited resources. Of the 10 women who were tested, however, only one was found to be seropositive for VEEV.

In horses, experimental infection with VEEV was associated with acute viremia, which was followed by death between 6 and 9 days. Clinical signs included blindness, wild behavior, circling, depression, fever, ulcers, leucopenia, reduced appetite, abnormal chewing movements, and a decrease in hematocrit counts^60,61^. Signs of viral encephalitis were detectable within 6 days after infection, with edema, focal hemorrhages, and neuronophagia found in fatal cases, accompanied by high viral loads in areas such as the cerebral cortex and cerebellum^60–62^. VEEV was readily detectable in body fluids such as saliva, blood, and feces^60^.

## Host immunity

Host adaptive immune responses have been shown to play a critical role in protection against VEEV infection, especially in cases of symptomatic infections, though the exact immune correlate of protection remains unclear. As with other arboviruses^63–66^, neutralizing antibodies (nAb) are thought to be a critical correlate of protection against VEEV infection. However, preclinical studies suggest that nAb responses may not be sufficient to prevent neurovirulence and CNS pathology in the absence of an effective T cell response^59,67–69^.

Paessler et al.^68^, examined the protective efficacy of a chimeric VEEV vaccine in B and T cell-deficient mice and compared them with wild-type (WT) mice. Vaccination was found to fully protect WT mice from lethal VEEV infection, whereas mice lacking the αβ T cell receptor (TCR) and a majority of μMT mice that lacked the immunoglobulin heavy chain experienced severe and lethal encephalitis, suggesting that T cells are essential for protection as compared to nAb responses. Passive transfer of VEEV-specific αβ+ T cells was found to protect αβ TCR-deficient mice from challenge. Likewise, Taylor et al.^59^, reported that αβ TCR-deficient mice experienced persistent CNS infection and inflammation following vaccination with the live-attenuated TC-83 vaccine, as compared to WT mice, suggesting that αβ TCR + T cells play a critical role in viral control. Other studies have suggested that CD4 + T cells are more effective at controlling VEEV infection in the CNS than CD8 + T cells^67,69^.

In contrast to the above studies, Kafai et al.^70^, demonstrated that a VEEV-specific monoclonal nAb isolated from mice and humans effectively blocked multiple steps in viral replication, including attachment, fusion, and viral release from infected cells, and protected these mice from infection following aerosol challenge with VEEV. Tretyakova et al.^71^, reported that non-human primates vaccinated with a live-attenuated VEEV vaccine engineered to prevent reversion induced high levels of VEEV-specific nAb, which correlated with a lack of viremia after aerosol challenge, as compared to unvaccinated animals. Other studies in NHP models have demonstrated the efficacy of VEEV-specific monoclonal nAb in significantly reducing viremia following VEEV challenge^67^. Interestingly, vaccination of B cell-deficient μMT mice with the live-attenuated TC-83 vaccine protected against aerosol challenge with VEEV only when combined with passive transfer of VEEV-specific antibodies, suggesting that both B and T cell responses were essential for protection against infection, neurovirulence, and neuropathogenesis^72^. Yun et al.^69^, reported that treatment of αβ TCR-deficient mice with VEEV-specific nAb prior to intranasal challenge was associated with increased survival rates, though it did not prevent neuropathogenesis and lethal encephalitis, whereas adoptive transfer of VEEV-specific CD4 + T cells protected these mice from severe encephalitis and mortality.

Taken together, these studies suggest that vaccines that can induce both VEEV-specific nAb and T cell responses could effectively prevent or clear CNS infections and protect against lethal disease and mortality associated with VEEV.

## Vaccine development and protective immunity

Given the lack of clearly defined correlates of protection, most preclinical and clinical vaccine development efforts have focused on inducing a combination of both potent nAb and T cell responses (Tables 1 and 2). There are currently no FDA-approved vaccines to protect against VEEV. However, a live-attenuated VEEV strain, TC-83, derived from the WT VEEV Trinidad Donkey (TrD) strain after repeated passaging through guinea pig heart cells, was approved for at-risk laboratory workers under FDA-approved protocols^73^. Attenuation was driven by a genetic mutation from guanine (G) to adenine (A) residue at position 3 in the 5′ UTR, along with an amino acid substitution from threonine to arginine at position 120 in the E2 envelope glycoprotein that attenuates replication and pathogenesis as compared to the prototypic WT TrD strain^74–76^. TC-83 was shown to produce smaller plaque sizes as compared to the TrD strain in vitro, and though both TC-83 and TrD show similar in vitro growth kinetics and cytopathic effects, the TrD strain has a higher capacity to replicate and produce more infectious virus^74,77^. TC-83 has been shown to be more sensitive to IFIT1, an interferon-stimulated gene, as compared to the TrD strain due to the mutation in the 5′ UTR that contributes to its in vivo attenuation^76,78^. Others have reported that TC-83 was cleared from the CNS by day 7 after infection in mice, whereas infection with the TrD strain was lethal at this timepoint^73^. The attenuated nature of TC-83 was the driving force behind its development as a potential vaccine candidate against VEEV.

**Table 1:** VEEV vaccines in clinical trials

Platform	Vaccine name	Phase	Clinical trial #	Known parameters and trial status
LAV	V4020	1	NCT07088822	**Current Status:** Not yet recruiting
IV	C-84	2	NCT03531242 NCT00582088	**Dosage:** 0. 1 ml (i.d) or 0.5 ml (s.c) of 9.5–10 log10 pfu/ml **Route:** Intradermal or subcutaneous **Safety:** Mild local and systemic reactions **Immunogenicity:** High titer nAb in non-immune subjects; boosted in subjects with pre-existing nAb titers^106^ **Current status:** Not yet recruiting (NCT03531242), Unknown (NCT00582088)
LAV	TC-83	2	NCT00582504 NCT03051386	**Dosage:** Single dose (10^5^ PFU) **Route:** Intradermal in trial 1 and subcutaneous in trial 2 **Safety:** AE in ~20% of vaccinees, virus shedding **Immunogenicity:** ~80% subjects had nAb titers^18^ **Current status:** Unknown
LAV	V3526	1	NCT00109304	**Dosage:** 125 and 25 PFU **Route:** Subcutaneous **Safety:** Mild to moderate AE, virus shedding **Immunogenicity:** High titer nAb titers **Current status:** Withdrawn
VLP	WEVEE	1	NCT03879603	**Dosage:** 2 doses of 6, 30, or 60 μg at week 0 and week 8 **Route:** Intramuscular **Safety:** Safe and well-tolerated, mild AE **Immunogenicity:** ~90% subjects had nAb titers^103^ **Current status:** Completed
DNA	pWRG/VEE	1	NCT01984983	**Dosage:** 0.5 mg, 2.0 mg (i.m.), 0.08 mg, 0.3 mg (i.d.) **Route:** Intramuscular or intradermal **Safety:** Safe and tolerable **Immunogenicity:** 100% in i.m., 62.5–87.5% i.d^105^. **Current status:** Completed

*IV* inactivated vaccine, *LAV* live-attenuated vaccine, *VLP* virus-like particle vaccine, *DNA* iDNA Vaccine, *i.m.* intramuscular, *i.d.* intradermal, *s.c.* subcutaneous.

**Table 2:** Selected preclinical live-attenuated VEE vaccine candidates

	Vaccine name	Vaccine description	Results from development
Medigen, USA	V4020	Two attenuating mutations from TC-83, E2-120 mutation genetically stabilized, genome rearranged, and subgenomic promoter duplicated	**Model:** Mice^107^ **Dosage:** 10^4^ PFU/mL **Route:** Subcutaneous **Safety:** No adverse reactions **Efficacy:** 100% protection **Model:** NHP^71^ **Dosage:** 10^4^ PFU **Route:** Subcutaneous, intramuscular **Safety:** No adverse reactions **Efficacy:** Challenge by aerosol, protection with reduced viremia.
UTMB, USA	SIN/VEE	Chimeric virus, nsP1-4 from Sindbis virus, C-E3-E2-6K-E1 from VEEV	**Model:** Mice^108^ **Dosage:** 5 × 10^5^ PFU **Route:** Subcutaneous **Safety:** Improved over TC-83 **Efficacy:** Protection observed.
UTMB, USA	VEEV/IRES 68U201/IRESv1	Subgenomic promoter replaced with IRES to express C-E3-E2-6K-E1	**Model:** NHP^95^ **Dosage:** 10^5^ PFU **Route:** Subcutaneous **Safety:** No adverse reactions. **Efficacy:** Inhaled challenge dose of 4 × 10^4^ pfu/animal, protection with reduced viremia.
UMD, USA	PRFm	Ablation of −1 programmed ribosomal frameshift between 6K and E1	**Model:** Mice^109^ **Dosage:** 10^5^ PFU/mL **Route:** Aerosol **Safety:** Attenuating effect of PRFm virus noted. **Efficacy:** ND

In vivo studies have demonstrated that the TC-83 vaccine induces a robust immune response in both animals and humans^17,73,79–81^. Experimental vaccination of horses with TC-83 was found to induce nAb responses in ~87% of vaccinated animals by 1-month post-vaccination, with responses persisting in ~73% of the animals at 1-year post-vaccination^80^. Likewise, a single subcutaneous dose of TC-83 vaccination in human laboratory workers induced PRNT_80_ titers of ≥1:20 in >80% of vaccinees. A subset of laboratory workers, including responders and non-responders, received a second vaccination with the formalin-fixed version of TC-83, known as C-84, and 76% of non-responders and 100% of responders exhibited PRNT_80_ titers of ≥1:20^17^. Interestingly, Fillis et al.^17^, reported that sera from laboratory workers vaccinated with the TC-83 vaccine displayed higher neutralization titers against the epidemic (IA, IB, and IC) strains of VEEV than against the endemic strains, suggesting that TC-83 vaccination did not likely induce high nAb titers against heterologous strains. However, TC-83 vaccination was found to protect mice against lethal infection from both epidemic and endemic strains of VEEV, suggesting that cellular immune responses may have contributed to protection against both strains of VEEV, as nAb profiles in mice were similar to those reported in humans^81^. Others have reported similar differences in the ability of sera from TC-83 vaccinees to neutralize the ID, IE, II, III, and IV subtypes of endemic VEEV strains^79^. TC-83 vaccination has been reported to induce mucosal B cell responses that correlated with protection from aerosol challenge when mice were challenged early after vaccination, compared with a late challenge^82–85^. Peripheral blood mononuclear cells obtained from TC-83-vaccinated human subjects displayed significant lymphocyte activation after in vitro stimulation with whole inactivated TC-83 virus, suggesting that vaccination induced a robust cellular immune response^86^. Others have reported that TC-83 vaccination was associated with activation of CD4 + T cells along with an IgG2a subtype, suggesting that vaccination induced a predominantly Th-1 type T cell response as compared to a more muted CD8 + T cell response^85,87,88^.

Since its initial use in a limited number of laboratory workers and human volunteers, adverse reactions ranging from headache, myalgia, malaise, sore throat, nausea, minor EKG abnormalities, fever, teratogenicity, and anorexia^17–19^ were reported in a number of TC-83 vaccinees. Others have suggested that the TC-83 vaccine virus could potentially be transmitted by mosquitoes, though there is limited evidence to support this hypothesis in humans. Pedersen et al.^89^, demonstrated that TC-83 could be isolated from mosquitoes found in areas surrounding equids that had been vaccinated with the TC-83 vaccine, whereas Turrell et al.^90^, demonstrated that TC-83 replicated in mosquitoes that could transmit the virus to hamsters, suggesting that mosquitoes could potentially spread the TC-83 infection within the human population. These factors, along with reports of poor immunogenicity in some volunteers and the potential for reversion to a lethal phenotype, have limited further clinical development of the TC-83 as an effective VEEV vaccine.

**Fig. 4:**
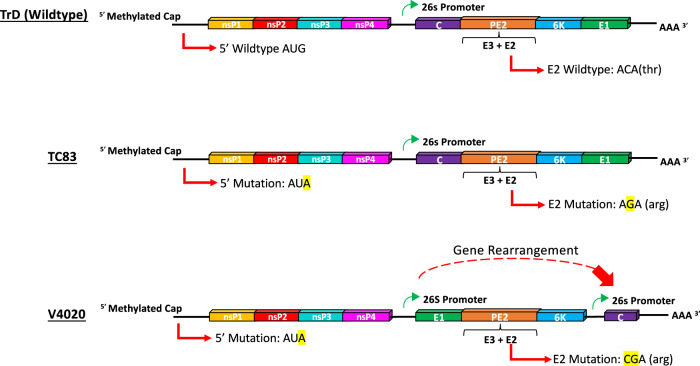
Cartoon illustration of live-attenuated vaccine genomes and attenuating features compared to the wild-type virus. TC-83 is a live-attenuated virus vaccine that was created by passaging the wild-type VEEV Trinidad Donkey (TrD) strain in guinea pig heart cells to attenuate the virus. The resulting attenuating features were found to be point mutations in the viral 5′ untranslated region (AUG → AUA) and the E2 encoding gene, the latter of which causing an amino acid substitution in E2 at position 120 (Threonine → Arginine). V4020 is also a live-attenuated vaccine derived from the TC-83 vaccine but contains additional, rationally designed attenuations. The introduction of a second point mutation in the E2 encoding gene (ACA → CGA instead of ACA → AGA) made reversion or pseudoreversion extremely unlikely compared to the TC-83 vaccine. Additionally, the structural genes have been rearranged, with the capsid moved to the 3’ end and placed under its own 26S promoter, which has previously been shown to attenuate viruses.

V4020 is a live-attenuated vaccine candidate derived from the TC-83 strain that was generated by engineering changes in the viral genome to prevent reversion by adding a second mutation in the E2 protein and rearranging the structural genes by moving the gene encoding for the capsid protein to the end of the genome (Fig. 4) without compromising immunogenicity. Cynomolgus macaques vaccinated with V4020 displayed significantly higher titers of VEEV-specific nAb responses without any adverse effects that correlated with protection from lethal aerosol challenge with VEEV^71^. Microneedle vaccination of rabbits with a single dose of V4020 vaccine induced VEEV-specific PRNT_80_ titers of 640 at day 21 post-vaccination with no signs of reactogenicity^91^. Centers et al.^92^, reported that mice vaccinated with V4020, either subcutaneously or intramuscularly, showed decreased reactogenicity compared with the parental TC-83 strain. Additionally, unlike the TC-83 vaccine, which was readily detectable in the cerebral cortex, cerebellum, and spinal cord of vaccinated mice at 6 days post-infection, V4020 was undetectable in the CNS of vaccinated mice, suggesting that V4020 had a significantly better safety profile than the TC-83 vaccine^92^. Likewise, Johnson et al.^93^, reported that intracranial inoculation of BALB/c mice with V4020 resulted in little or no replication and lower levels of inflammation compared with the TC-83 vaccine. Additionally, the V4020 vaccine remained safe even after 5 passages in mouse brains, compared with TC-83, which became lethal after 3 passages, suggesting greater attenuation of V4020^93^. Taken together, the above studies suggest that the V4020 vaccine is a novel and potentially safe alternative to the TC-83 vaccine that could induce complete immunity and protect against a lethal challenge.

In addition to the V4020 vaccine, several other experimental vaccine candidates have been developed to overcome the limitations associated with the TC-83 vaccine. Rossi et al. demonstrated that 68U201/IRES1, a vaccine derived from an attenuated endemic strain (IE) of VEEV by inserting an internal ribosomal entry site (IRES) upstream of the structural polyprotein open reading frame, protected mice against lethal challenge and protected NHP against febrile disease after a homologous challenge^94,95^. Notably, 68U201/IRES1 failed to productively infect mosquitoes, suggesting that the vaccine virus could not be transmitted by mosquitoes to others^94^. Chen et al. described a replicon vaccine encoding the structural proteins of VEEV, WEEV, and EEEV that protected mice from aerosol and subcutaneous challenge, though its efficacy was somewhat limited in the NHP model^96^. The V3526 live-attenuated vaccine was derived from the VEEV-IA/B TrD strain through mutagenesis of the furin cleavage site, along with a second mutation in the E1 protein, which was shown to protect both NHP and mice from aerosol challenge with IA/B and IE strains of VEEV^97–99^. Additionally, Turrel et al.^100^, demonstrated that V3526 vaccinated hamsters resisted lethal TrD infection transmitted by *Aedes taeniorhynchus* that were inoculated with the TrD strain of VEEV. On the other hand, Rao et al. reported that the adverse impact of V3526 on public health and the environment, and the potential for transmission were low^101^. A chimeric vaccine derived from Sindbis virus (family *Togaviridae*, genus *Alphavirus)* that expressed structural proteins from different VEEV strains was reported to protect mice and hamsters from lethal challenge^102^.

**Fig. 5:**
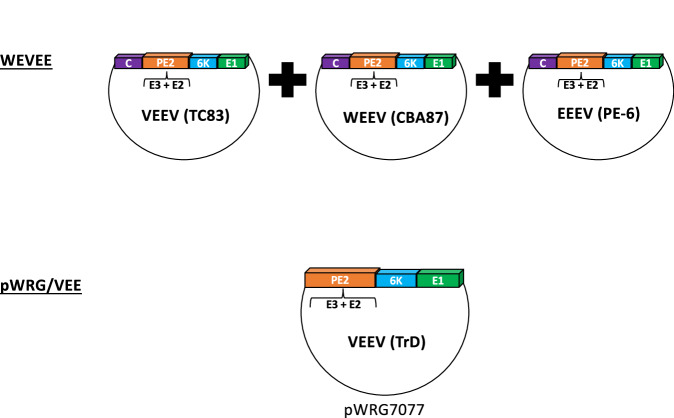
Cartoon illustration of genomes used for the WEVEE and pWRG/VEE vaccine. WEVEE is a mix of EEEV, WEEV, and VEEV VLP’s encoding the structural genes (C-E3-E2-6K-E1) for each virus. Similarly, the pWRG/VEE vaccine is a DNA vaccine based on the pWRG7077 backbone that encodes the VEEV structural proteins E3, E2, 6 K, and E1.

The WEVEE vaccine (VRC-WEVVLP073-00-VP) is a virus-like particle (VLP) vaccine that combines the VLPs for EEEV, WEEV, and VEEV, encoding the structural genes (C-E3-E2-6K-E1) for each virus (Fig. 5). In Phase 1 clinical trials (NCT03879603), the vaccine was found to be safe and induced nAb responses against all three viruses^103^. The pWRG/VEE vaccine is a DNA vaccine encoding the structural genes E3, E2, 6K, and E1 of VEEV and has been demonstrated to be efficacious in both mice and rabbits, and in reducing morbidity and viremia in the NHP model^104^. In Phase 1 trials (NCT01984983), the vaccine was found to be safe and induced VEEV-specific nAb responses^105^.

In summary, VEEV remains a major concern given its potential for large-scale outbreaks. The TC-83 vaccine is the only vaccine approved for limited use among laboratory workers. However, concerns of variable immunogenicity, potential for reversion, and adverse reactions have limited further clinical development of the TC-83 vaccine. There remains an urgent need to develop a safe and efficacious vaccine for human use. Novel vaccines such as the V4020, V3526, along with the VLP-based WEVEE and pWRG/VEE vaccines have been shown to be highly immunogenic and efficacious, suggesting that these vaccines, if successful, could potentially fill a critical clinical need.

## Data Availability

No datasets were generated or analysed during the current study.

## References

[1] Forrester, N. L. et al. Evolution and spread of Venezuelan equine encephalitis complex alphavirus in the Americas. *PLoS Negl. Trop. Dis.* **11**, e0005693 (2017). 28771475 10.1371/journal.pntd.0005693 PMC5557581

[2] Estrada-Franco, J. G. et al. Venezuelan equine encephalitis virus, southern Mexico. *Emerg. Infect. Dis.* **10**, 2113–2121 (2004). 15663847 10.3201/eid1012.040393 PMC3323369

[3] Kubes, V. & Rios, F. A. The causative agent of infectious equine encephalomyelitis in Venezuela. *Science* **90**, 20–21 (1939). 17818578 10.1126/science.90.2323.20

[4] Oberste, M. S. et al. Association of Venezuelan equine encephalitis virus subtype IE with two equine epizootics in Mexico. *Am. J. Trop. Med. Hyg.* **59**, 100–107 (1998). 9684636 10.4269/ajtmh.1998.59.100

[5] Barrera, R. et al. Contrasting sylvatic foci of Venezuelan equine encephalitis virus in northern South America. *Am. J. Trop. Med. Hyg.* **67**, 324–334 (2002). 12408676 10.4269/ajtmh.2002.67.324

[6] Brault, A. C., Powers, A. M. & Weaver, S. C. Vector infection determinants of Venezuelan equine encephalitis virus reside within the E2 envelope glycoprotein. *J. Virol.* **76**, 6387–6392 (2002). 12021373 10.1128/JVI.76.12.6387-6392.2002 PMC136209

[7] Ferro, C. et al. Natural enzootic vectors of Venezuelan equine encephalitis virus, Magdalena Valley, Colombia. *Emerg. Infect. Dis.* **9**, 49–54 (2003). 12533281 10.3201/eid0901.020136 PMC2873762

[8] Kramer, L. D. & Scherer, W. F. Vector competence of mosquitoes as a marker to distinguish Central American and Mexican epizootic from enzootic strains of Venezuelan encephalitis virus. *Am. J. Trop. Med. Hyg.* **25**, 336–346 (1976). 1259093 10.4269/ajtmh.1976.25.336

[9] Navarro, J. C. & Weaver, S. C. Molecular phylogeny of the Vomerifer and Pedroi Groups in the Spissipes Section of the subgenus Culex (Melanoconion). *J. Med. Entomol.* **41**, 575–581 (2004). 15311446 10.1603/0022-2585-41.4.575

[10] Rivas, F. et al. Epidemic Venezuelan equine encephalitis in La Guajira, Colombia, 1995. *J. Infect. Dis.* **175**, 828–832 (1997). 9086137 10.1086/513978

[11] Franz, D. R. et al. Clinical recognition and management of patients exposed to biological warfare agents. *Clin. Lab Med.* **21**, 435–473 (2001). 11572137

[12] Hanson, R. P. et al. Arbovirus infections of laboratory workers. Extent of problem emphasizes the need for more effective measures to reduce hazards. *Science* **158**, 1283–1286 (1967). 6058003 10.1126/science.158.3806.1283

[13] Phelps, A. L. et al. Susceptibility and lethality of western equine encephalitis virus in Balb/c mice when infected by the aerosol route. *Viruses* **9**, 10.3390/v9070163 (2017). 10.3390/v9070163 PMC5537655 28654007

[14] Reed, D. S. et al. Aerosol exposure to western equine encephalitis virus causes fever and encephalitis in cynomolgus macaques. *J. Infect. Dis.* **192**, 1173–1182 (2005). 16136459 10.1086/444397

[15] Roy, C. J. et al. Pathogenesis of aerosolized Eastern Equine Encephalitis virus infection in guinea pigs. *Virol. J.* **6**, 170 (2009). 19852817 10.1186/1743-422X-6-170 PMC2770496

[16] Williams, J. A. et al. Eastern equine encephalitis virus rapidly infects and disseminates in the brain and spinal cord of cynomolgus macaques following aerosol challenge. *PLoS Negl. Trop. Dis.* **16**, e0010081 (2022). 35533188 10.1371/journal.pntd.0010081 PMC9084534

[17] Pittman, P. R. et al. Long-term duration of detectable neutralizing antibodies after administration of live-attenuated VEE vaccine and following booster vaccination with inactivated VEE vaccine. *Vaccine* **14**, 337–343 (1996). 8744562 10.1016/0264-410x(95)00168-z

[18] Alevizatos, A. C., McKinney, R. W. & Feigin, R. D. Live, attenuated Venezuelan equine encephalomyelitis virus vaccine. *Clin. Eff. Man. Am. J. Trop. Med. Hyg.* **16**, 762–768 (1967). 6066224

[19] Casamassima, A. C., Hess, L. W. & Marty, A. TC-83 Venezuelan equine encephalitis vaccine exposure during pregnancy. *Teratology* **36**, 287–289 (1987). 3424216 10.1002/tera.1420360303

[20] Guzman-Teran, C., Calderon-Rangel, A., Rodriguez-Morales, A. & Mattar, S. Venezuelan equine encephalitis virus: the problem is not over for tropical America. *Ann. Clin. Microbiol. Antimicrob.* **19**, 19 (2020). 32429942 10.1186/s12941-020-00360-4 PMC7236962

[21] Luethy, D. Eastern, Western, and Venezuelan Equine Encephalitis and West Nile viruses: clinical and public health considerations. *Vet. Clin. North Am. Equine Pr.* **39**, 99–113 (2023). 10.1016/j.cveq.2022.11.007 36737290

[22] Weaver, S. C., Bellew, L. A. & Rico-Hesse, R. Phylogenetic analysis of alphaviruses in the Venezuelan equine encephalitis complex and identification of the source of epizootic viruses. *Virology* **191**, 282–290 (1992). 1413507 10.1016/0042-6822(92)90190-z

[23] Anishchenko, M. et al. Venezuelan encephalitis emergence mediated by a phylogenetically predicted viral mutation. *Proc. Natl. Acad. Sci. USA* **103**, 4994–4999 (2006). 16549790 10.1073/pnas.0509961103 PMC1458783

[24] Moncayo, A. C. et al. Vector competence of eastern and western forms of Psorophora columbiae (Diptera: Culicidae) mosquitoes for enzootic and epizootic Venezuelan equine encephalitis virus. *Am. J. Trop. Med. Hyg.* **78**, 413–421 (2008). 18337337

[25] Ortiz, D. I., Anishchenko, M. & Weaver, S. C. Susceptibility of Psorophora confinnis (Diptera: Culicidae) to infection with epizootic (subtype IC) and enzootic (subtype ID) Venezuelan Equine encephalitis viruses. *J. Med. Entomol.* **42**, 857–863 (2005). 16365999 10.1093/jmedent/42.5.857

[26] Sudia, W. D. et al. Epidemic Venezuelan equine encephalitis in North America in 1971: vector studies. *Am. J. Epidemiol.* **101**, 17–35 (1975). 235212 10.1093/oxfordjournals.aje.a112068

[27] Kafai, N. M. et al. Entry receptor LDLRAD3 is required for Venezuelan equine encephalitis virus peripheral infection and neurotropism leading to pathogenesis in mice. *Cell Rep.* **42**, 112946 (2023). 37556325 10.1016/j.celrep.2023.112946 PMC10529316

[28] Ma, B., Huang, C., Ma, J., Xiang, Y. & Zhang, X. Structure of Venezuelan equine encephalitis virus with its receptor LDLRAD3. *Nature* **598**, 677–681 (2021). 34646021 10.1038/s41586-021-03909-1

[29] Ma, H. et al. LDLRAD3 is a receptor for Venezuelan equine encephalitis virus. *Nature* **588**, 308–314 (2020). 33208938 10.1038/s41586-020-2915-3 PMC7769003

[30] Adeyinka, O. S. et al. nsP2 protease inhibitor blocks the replication of New World alphaviruses and offer protection in mice. *ACS Infect. Dis.* **11**, 181–196 (2025). 39737550 10.1021/acsinfecdis.4c00701

[31] de Groot, R. J., Hardy, W. R., Shirako, Y. & Strauss, J. H. Cleavage-site preferences of Sindbis virus polyproteins containing the non-structural proteinase. Evidence for temporal regulation of polyprotein processing in vivo. *EMBO J.* **9**, 2631–2638 (1990). 2142454 10.1002/j.1460-2075.1990.tb07445.x PMC552296

[32] Lark, T., Keck, F. & Narayanan, A. Interactions of alphavirus nsP3 protein with host proteins. *Front. Microbiol.* **8**, 2652 (2017). 29375517 10.3389/fmicb.2017.02652 PMC5767282

[33] Shirako, Y. & Strauss, J. H. Regulation of Sindbis virus RNA replication: uncleaved P123 and nsP4 function in minus-strand RNA synthesis, whereas cleaved products from P123 are required for efficient plus-strand RNA synthesis. *J. Virol.* **68**, 1874–1885 (1994). 8107248 10.1128/jvi.68.3.1874-1885.1994 PMC236650

[34] Li, C. et al. mRNA capping by Venezuelan equine encephalitis virus nsP1: functional characterization and implications for antiviral research. *J. Virol.* **89**, 8292–8303 (2015). 26041283 10.1128/JVI.00599-15 PMC4524220

[35] Hoffka, G. et al. Self-inhibited state of Venezuelan equine encephalitis virus (VEEV) nsP2 cysteine protease: a crystallographic and molecular dynamics analysis. *J. Mol. Biol.* **435**, 168012 (2023). 36792007 10.1016/j.jmb.2023.168012 PMC10758287

[36] Kim, D. Y., Atasheva, S., Frolova, E. I. & Frolov, I. Venezuelan equine encephalitis virus nsP2 protein regulates packaging of the viral genome into infectious virions. *J. Virol.* **87**, 4202–4213 (2013). 23365438 10.1128/JVI.03142-12 PMC3624340

[37] Foy, N. J., Akhrymuk, M., Shustov, A. V., Frolova, E. I. & Frolov, I. Hypervariable domain of nonstructural protein nsP3 of Venezuelan equine encephalitis virus determines cell-specific mode of virus replication. *J. Virol.* **87**, 7569–7584 (2013). 23637407 10.1128/JVI.00720-13 PMC3700263

[38] Kril, V. et al. Alphavirus nsP3 organizes into tubular scaffolds essential for infection and the cytoplasmic granule architecture. *Nat. Commun.* **15**, 8106 (2024). 39285216 10.1038/s41467-024-51952-z PMC11405681

[39] Tan, Y. B. et al. Crystal structures of alphavirus nonstructural protein 4 (nsP4) reveal an intrinsically dynamic RNA-dependent RNA polymerase fold. *Nucleic Acids Res.* **50**, 1000–1016 (2022). 35037043 10.1093/nar/gkab1302 PMC8789068

[40] Kielian, M., Chanel-Vos, C. & Liao, M. Alphavirus entry and membrane fusion. *Viruses* **2**, 796–825 (2010). 21546978 10.3390/v2040796 PMC3086016

[41] Leung, J. Y., Ng, M. M. & Chu, J. J. Replication of alphaviruses: a review on the entry process of alphaviruses into cells. *Adv. Virol.* **2011**, 249640 (2011). 22312336 10.1155/2011/249640 PMC3265296

[42] Mukhopadhyay, S. et al. Mapping the structure and function of the E1 and E2 glycoproteins in alphaviruses. *Structure* **14**, 63–73 (2006). 16407066 10.1016/j.str.2005.07.025 PMC2757649

[43] Mayne, J. T., Rice, C. M., Strauss, E. G., Hunkapiller, M. W. & Strauss, J. H. Biochemical studies of the maturation of the small Sindbis virus glycoprotein E3. *Virology* **134**, 338–357 (1984). 6443592 10.1016/0042-6822(84)90302-7

[44] Sjoberg, M., Lindqvist, B. & Garoff, H. Activation of the alphavirus spike protein is suppressed by bound E3. *J. Virol.* **85**, 5644–5650 (2011). 21430054 10.1128/JVI.00130-11 PMC3094962

[45] Liljestrom, P. & Garoff, H. Internally located cleavable signal sequences direct the formation of Semliki Forest virus membrane proteins from a polyprotein precursor. *J. Virol.* **65**, 147–154 (1991). 1985194 10.1128/jvi.65.1.147-154.1991 PMC240499

[46] Welch, W. J. & Sefton, B. M. Two small virus-specific polypeptides are produced during infection with Sindbis virus. *J. Virol.* **29**, 1186–1195 (1979). 448798 10.1128/jvi.29.3.1186-1195.1979 PMC353279

[47] Zhang, R. et al. 4.4 A cryo-EM structure of an enveloped alphavirus Venezuelan equine encephalitis virus. *EMBO J.* **30**, 3854–3863 (2011). 21829169 10.1038/emboj.2011.261 PMC3173789

[48] Parker, M. D. et al. Antibody to the E3 glycoprotein protects mice against lethal venezuelan equine encephalitis virus infection. *J. Virol.* **84**, 12683–12690 (2010). 20926570 10.1128/JVI.01345-10 PMC3004303

[49] Atasheva, S., Garmashova, N., Frolov, I. & Frolova, E. Venezuelan equine encephalitis virus capsid protein inhibits nuclear import in Mammalian but not in mosquito cells. *J. Virol.* **82**, 4028–4041 (2008). 18256144 10.1128/JVI.02330-07 PMC2293000

[50] Negi, V., Miller, A. S. & Kuhn, R. J. Advances in viroporin function and structure: a comparative analysis of alphavirus 6k with well-characterized viroporins. *Viruses* **17**, 868 (2025). 10.3390/v17060868 PMC12197650 40573459

[51] Gardner, C. L. et al. Eastern and Venezuelan equine encephalitis viruses differ in their ability to infect dendritic cells and macrophages: impact of altered cell tropism on pathogenesis. *J. Virol.* **82**, 10634–10646 (2008). 18768986 10.1128/JVI.01323-08 PMC2573165

[52] Trabalza, A. et al. Venezuelan equine encephalitis virus glycoprotein pseudotyping confers neurotropism to lentiviral vectors. *Gene Ther.* **20**, 723–732 (2013). 23171919 10.1038/gt.2012.85

[53] Hollidge, B. S. et al. Toll-like receptor 4 mediates blood-brain barrier permeability and disease in C3H mice during Venezuelan equine encephalitis virus infection. *Virulence* **12**, 430–443 (2021). 33487119 10.1080/21505594.2020.1870834 PMC7849679

[54] Salimi, H. et al. Encephalitic alphaviruses exploit caveola-mediated transcytosis at the blood-brain barrier for central nervous system entry. *mBio* **11**, 10.1128/mBio.02731-19 (2020). 10.1128/mBio.02731-19 PMC7018649 32047126

[55] Charles, P. C., Walters, E., Margolis, F. & Johnston, R. E. Mechanism of neuroinvasion of Venezuelan equine encephalitis virus in the mouse. *Virology* **208**, 662–671 (1995). 7747437 10.1006/viro.1995.1197

[56] Weaver, S. C. et al. Re-emergence of epidemic Venezuelan equine encephalomyelitis in South America. *VEE Study Group. Lancet* **348**, 436–440 (1996). 10.1016/s0140-6736(96)02275-1 8709783

[57] Aguilar, P. V. et al. Endemic Venezuelan equine encephalitis in the Americas: hidden under the dengue umbrella. *Future Virol.* **6**, 721–740 (2011). 21765860 10.2217/FVL.11.5 PMC3134406

[58] Steele, K. E. & Twenhafel, N. A. REVIEW PAPER: pathology of animal models of alphavirus encephalitis. *Vet. Pathol.* **47**, 790–805 (2010). 20551475 10.1177/0300985810372508

[59] Taylor, K., Kolokoltsova, O., Ronca, S. E., Estes, M. & Paessler, S. Live, attenuated Venezuelan equine encephalitis virus vaccine (TC83) causes persistent brain infection in mice with non-functional alphabeta T-Cells. *Front. Microbiol.* **8**, 81 (2017). 28184218 10.3389/fmicb.2017.00081 PMC5266681

[60] Henderson, B. E., Chappell, W. A., Johnston, J. G. Jr & Sudia, W. D. Experimental infection of horses with three strains of Venezuelan equine encephalomyelitis virus. I. Clinical and virological studies. *Am. J. Epidemiol.* **93**, 194–205 (1971). 4397564 10.1093/oxfordjournals.aje.a121246

[61] Mackenzie, R. M., de Siger, J. & Parra, D. Venezuelan equine encephalitis virus: comparison of infectivity and virulence of strains V-38 and P676 in donkeys. *Am. J. Trop. Med. Hyg.* **25**, 494–499 (1976). 937636 10.4269/ajtmh.1976.25.494

[62] Greene, I. P. et al. Envelope glycoprotein mutations mediate equine amplification and virulence of epizootic Venezuelan equine encephalitis virus. *J. Virol.* **79**, 9128–9133 (2005). 15994807 10.1128/JVI.79.14.9128-9133.2005 PMC1168750

[63] George, J. et al. Prior exposure to Zika virus significantly enhances peak dengue-2 viremia in rhesus macaques. *Sci. Rep.* **7**, 10498 (2017). 28874759 10.1038/s41598-017-10901-1 PMC5585353

[64] Valiant, W. G. et al. Zika convalescent macaques display delayed induction of anamnestic cross-neutralizing antibody responses after dengue infection. *Emerg. Microbes Infect.* **7**, 130 (2018). 30006514 10.1038/s41426-018-0132-z PMC6045599

[65] Valiant, W. G. et al. Human serum with high neutralizing antibody titres against both Zika and dengue virus shows delayed in vitro antibody dependent enhancement of dengue virus infection. *Open Forum Infect. Dis.* **5**, ofy151 (2018). 10.1093/ofid/ofy151 PMC6041987 30019003

[66] Valiant, W. G. et al. Simultaneous coinfection of macaques with Zika and dengue viruses does not enhance acute plasma viremia but leads to activation of monocyte subsets and biphasic release of pro-inflammatory cytokines. *Sci. Rep.* **9**, 7877 (2019). 31133721 10.1038/s41598-019-44323-y PMC6536518

[67] Burke, C. W. et al. Therapeutic monoclonal antibody treatment protects nonhuman primates from severe Venezuelan equine encephalitis virus disease after aerosol exposure. *PLoS Pathog.* **15**, e1008157 (2019). 31790515 10.1371/journal.ppat.1008157 PMC6907853

[68] Paessler, S. et al. Alpha-beta T cells provide protection against lethal encephalitis in the murine model of VEEV infection. *Virology* **367**, 307–323 (2007). 17610927 10.1016/j.virol.2007.05.041 PMC2067255

[69] Yun, N. E. et al. CD4 + T cells provide protection against acute lethal encephalitis caused by Venezuelan equine encephalitis virus. *Vaccine* **27**, 4064–4073 (2009). 19446933 10.1016/j.vaccine.2009.04.015 PMC2741389

[70] Kafai, N. M. et al. Neutralizing antibodies protect mice against Venezuelan equine encephalitis virus aerosol challenge. *J. Exp. Med.* **219**, 10.1084/jem.20212532 (2022). 10.1084/jem.20212532 PMC9195047 35297953

[71] Tretyakova, I. et al. Venezuelan equine encephalitis vaccine with rearranged genome resists reversion and protects non-human primates from viremia after aerosol challenge. *Vaccine* **38**, 3378–3386 (2020). 32085953 10.1016/j.vaccine.2020.02.007

[72] Elvin, S. J., Bennett, A. M. & Phillpotts, R. J. Role for mucosal immune responses and cell-mediated immune functions in protection from airborne challenge with Venezuelan equine encephalitis virus. *J. Med. Virol.* **67**, 384–393 (2002). 12116032 10.1002/jmv.10086

[73] Berge, T. O. B., I. S. & Tigertt, W. D. Attenuation of Venezuelan equine encephalomyelitis virus by in vitro cultivation in guinea-pig heart cells. *Am. J. Epidemiol.* **73**, 209–218 (1961).

[74] Kinney, R. M. et al. Attenuation of Venezuelan equine encephalitis virus strain TC-83 is encoded by the 5’-noncoding region and the E2 envelope glycoprotein. *J. Virol.* **67**, 1269–1277 (1993). 7679745 10.1128/jvi.67.3.1269-1277.1993 PMC237493

[75] Kinney, R. M., Johnson, B. J., Welch, J. B., Tsuchiya, K. R. & Trent, D. W. The full-length nucleotide sequences of the virulent Trinidad donkey strain of Venezuelan equine encephalitis virus and its attenuated vaccine derivative, strain TC-83. *Virology* **170**, 19–30 (1989). 2524126 10.1016/0042-6822(89)90347-4

[76] White, L. J., Wang, J. G., Davis, N. L. & Johnston, R. E. Role of alpha/beta interferon in Venezuelan equine encephalitis virus pathogenesis: effect of an attenuating mutation in the 5’ untranslated region. *J. Virol.* **75**, 3706–3718 (2001). 11264360 10.1128/JVI.75.8.3706-3718.2001 PMC114862

[77] Mecham, J. O. & Trent, D. W. A biochemical comparison of the in vitro replication of a virulent and an avirulent strain of Venezuelan encephalitis virus. *J. Gen. Virol.* **64**, 1111–1119 (1983). 6842187 10.1099/0022-1317-64-5-1111

[78] Hyde, J. L. et al. A viral RNA structural element alters host recognition of nonself RNA. *Science* **343**, 783–787 (2014). 24482115 10.1126/science.1248465 PMC4209899

[79] Burke, D. S., Ramsburg, H. H. & Edelman, R. Persistence in humans of antibody to subtypes of Venezuelan equine encephalomyelitis (VEE) virus after immunization with attenuated (TC-83) VEE virus vaccine. *J. Infect. Dis.* **136**, 354–359 (1977). 903673 10.1093/infdis/136.3.354

[80] Ferguson, J. A., Reeves, W. C., Milby, M. M. & Hardy, J. L. Study of homologous and heterologous antibody response in California horses vaccinated with attenuated Venezuelan equine encephalomyelitis vaccine (strain TC-83). *Am. J. Vet. Res.* **39**, 371–376 (1978). 637386

[81] Fillis, C. A. & Calisher, C. H. Neutralizing antibody responses of humans and mice to vaccination with Venezuelan encephalitis (TC-83) virus. *J. Clin. Microbiol.* **10**, 544–549 (1979). 93607 10.1128/jcm.10.4.544-549.1979 PMC273212

[82] Jahrling, P. B. & Stephenson, E. H. Protective efficacies of live attenuated and formaldehyde-inactivated Venezuelan equine encephalitis virus vaccines against aerosol challenge in hamsters. *J. Clin. Microbiol.* **19**, 429–431 (1984). 6715512 10.1128/jcm.19.3.429-431.1984 PMC271080

[83] Hart, M. K., Pratt, W., Panelo, F., Tammariello, R. & Dertzbaugh, M. Venezuelan equine encephalitis virus vaccines induce mucosal IgA responses and protection from airborne infection in BALB/c, but not C3H/HeN mice. *Vaccine* **15**, 363–369 (1997). 9141206 10.1016/s0264-410x(96)00204-6

[84] Phillpotts, R. J. & Wright, A. J. TC-83 vaccine protects against airborne or subcutaneous challenge with heterologous mouse-virulent strains of Venezuelan equine encephalitis virus. *Vaccine* **17**, 982–988 (1999). 10067707 10.1016/s0264-410x(98)00315-6

[85] Bennett, A. M., Elvin, S. J., Wright, A. J., Jones, S. M. & Phillpotts, R. J. An immunological profile of Balb/c mice protected from airborne challenge following vaccination with a live attenuated Venezuelan equine encephalitis virus vaccine. *Vaccine* **19**, 337–347 (2000). 10930689 10.1016/s0264-410x(00)00123-7

[86] Marker, S. C. & Ascher, M. S. Specific in vitro lymphocyte transformation with Venezuelan equine encephalitis virus. *Cell Immunol.* **23**, 32–38 (1976). 57835 10.1016/0008-8749(76)90169-6

[87] Jones, L. D., Bennett, A. M., Moss, S. R., Gould, E. A. & Phillpotts, R. J. Cytotoxic T-cell activity is not detectable in Venezuelan equine encephalitis virus-infected mice. *Virus Res.* **91**, 255–259 (2003). 12573505 10.1016/s0168-1702(02)00275-7

[88] Mathews, J. H., Kinney, R. M., Roehrig, J. T., Barrett, A. D. & Trent, D. W. Murine T-helper cell immune response to recombinant vaccinia-Venezuelan equine encephalitis virus. *Vaccine* **12**, 620–624 (1994). 8085379 10.1016/0264-410x(94)90266-6

[89] Pedersen, C. E. Jr, Robinson, D. M. & Cole, F. E. Jr. Isolation of the vaccine strain of Venezuelan equine encephalomyelitis virus from mosquitoes in Louisiana. *Am. J. Epidemiol.* **95**, 490–496 (1972). 4401801 10.1093/oxfordjournals.aje.a121416

[90] Turell, M. J., Ludwig, G. V., Kondig, J. & Smith, J. F. Limited potential for mosquito transmission of genetically engineered, live-attenuated Venezuelan equine encephalitis virus vaccine candidates. *Am. J. Trop. Med. Hyg.* **60**, 1041–1044 (1999). 10403340 10.4269/ajtmh.1999.60.1041

[91] Tretyakova, I., Tomai, M., Vasilakos, J. & Pushko, P. Live-attenuated VEEV vaccine delivered by iDNA using microneedles is immunogenic in rabbits. *Front. Trop. Dis.* **3**, 10.3389/fitd.2022.813671 (2022). 10.3389/fitd.2022.813671 PMC10583749 37854093

[92] Centers, A. et al. V4020 Venezuelan equine encephalitis vaccine: mitigating neuroinvasion and reversion through rational design. *Viruses* **17**, 10.3390/v17081136 (2025). 10.3390/v17081136 PMC12390694 40872849

[93] Johnson, D. M. et al. Advanced safety and genetic stability in mice of a novel DNA-launched Venezuelan equine encephalitis virus vaccine with rearranged structural genes. *Vaccines* **8**, 10.3390/vaccines8010114 (2020). 10.3390/vaccines8010114 PMC7157698 32121666

[94] Rossi, S. L. et al. IRES-based Venezuelan equine encephalitis vaccine candidate elicits protective immunity in mice. *Virology* **437**, 81–88 (2013). 23351391 10.1016/j.virol.2012.11.013 PMC3767167

[95] Rossi, S. L. et al. IRES-containing VEEV vaccine protects cynomolgus macaques from IE Venezuelan equine encephalitis virus aerosol challenge. *PLoS Negl. Trop. Dis.* **9**, e0003797 (2015). 26020513 10.1371/journal.pntd.0003797 PMC4447396

[96] Reed, D. S. et al. Combined alphavirus replicon particle vaccine induces durable and cross-protective immune responses against equine encephalitis viruses. *J. Virol.* **88**, 12077–12086 (2014). 25122801 10.1128/JVI.01406-14 PMC4178741

[97] Hart, M. K. et al. Improved mucosal protection against Venezuelan equine encephalitis virus is induced by the molecularly defined, live-attenuated V3526 vaccine candidate. *Vaccine* **18**, 3067–3075 (2000). 10825611 10.1016/s0264-410x(00)00042-6

[98] Pratt, W. D., Davis, N. L., Johnston, R. E. & Smith, J. F. Genetically engineered, live attenuated vaccines for Venezuelan equine encephalitis: testing in animal models. *Vaccine* **21**, 3854–3862 (2003). 12922119 10.1016/s0264-410x(03)00328-1

[99] Reed, D. S. et al. Genetically engineered, live, attenuated vaccines protect nonhuman primates against aerosol challenge with a virulent IE strain of Venezuelan equine encephalitis virus. *Vaccine* **23**, 3139–3147 (2005). 15837213 10.1016/j.vaccine.2004.12.023

[100] Turell, M. J. & Parker, M. D. Protection of hamsters by Venezuelan equine encephalitis virus candidate. *Am. J. Trop. Med. Hyg.* **78**, 328–332 (2008). 18256440

[101] Rao, V., Hinz, M. E., Roberts, B. A. & Fine, D. Environmental hazard assessment of Venezuelan equine encephalitis virus vaccine candidate strain V3526. *Vaccine* **22**, 2667–2673 (2004). 15193393 10.1016/j.vaccine.2003.09.041

[102] Paessler, S. et al. Replication and clearance of Venezuelan equine encephalitis virus from the brains of animals vaccinated with chimeric SIN/VEE viruses. *J. Virol.* **80**, 2784–2796 (2006). 16501087 10.1128/JVI.80.6.2784-2796.2006 PMC1395430

[103] Coates, E. E. et al. Safety and immunogenicity of a trivalent virus-like particle vaccine against western, eastern, and Venezuelan equine encephalitis viruses: a phase 1, open-label, dose-escalation, randomised clinical trial. *Lancet Infect. Dis.* **22**, 1210–1220 (2022). 35568049 10.1016/S1473-3099(22)00052-4 PMC9329218

[104] Dupuy, L. C. et al. A DNA vaccine for Venezuelan equine encephalitis virus delivered by intramuscular electroporation elicits high levels of neutralizing antibodies in multiple animal models and provides protective immunity to mice and nonhuman primates. *Clin. Vaccin. Immunol.* **18**, 707–716 (2011). 10.1128/CVI.00030-11 PMC3122536 21450977

[105] Hannaman, D., Dupuy, L. C., Ellefsen, B. & Schmaljohn, C. S. A Phase 1 clinical trial of a DNA vaccine for Venezuelan equine encephalitis delivered by intramuscular or intradermal electroporation. *Vaccine* **34**, 3607–3612 (2016). 27206386 10.1016/j.vaccine.2016.04.077

[106] Edelman, R. et al. Evaluation in humans of a new, inactivated vaccine for Venezuelan equine encephalitis virus (C-84). *J. Infect. Dis.* **140**, 708–715 (1979). 528788 10.1093/infdis/140.5.708

[107] Tretyakova, I. et al. Novel DNA-launched Venezuelan equine encephalitis virus vaccine with rearranged genome. *Vaccine* **37**, 3317–3325 (2019). 31072736 10.1016/j.vaccine.2019.04.072

[108] Paessler, S. & Weaver, S. C. Vaccines for Venezuelan equine encephalitis. *Vaccine* **27**, D80–D85 (2009). 19837294 10.1016/j.vaccine.2009.07.095 PMC2764542

[109] Kendra, J. A. et al. Ablation of programmed -1 ribosomal frameshifting in Venezuelan equine encephalitis virus results in attenuated neuropathogenicity. *J. Virol.* **91**, 10.1128/JVI.01766-16 (2017). 10.1128/JVI.01766-16 PMC5244343 27852852

